# Supplementary material for: Modeling glioblastoma heterogeneity as a dynamic network of cell states
Source: Mol Syst Biol. 2021 Sep 16;17(9):e10105. doi: 10.15252/msb.202010105 (PMC8444284; doi:10.15252/msb.202010105)
Supplement: Supplementary file 6 — Source Data for Figure 5 [file MSB-17-e10105-s004.zip › Figure5A_sourcedata/GSEA_3071/hallmarks_stateV.GseaPreranked.1623742546329/HALLMARK_MTORC1_SIGNALING.html]

Details for gene set HALLMARK\_MTORC1\_SIGNALING[GSEA]

|  || Dataset | state43071 |
| Phenotype | NoPhenotypeAvailable |
| Upregulated in class | na\_neg |
| GeneSet | HALLMARK\_MTORC1\_SIGNALING |
| Enrichment Score (ES) | -0.3445636 |
| Normalized Enrichment Score (NES) | -1.9370825 |
| Nominal p-value | 0.0 |
| FDR q-value | 0.0036569238 |
| FWER p-Value | 0.036 |
Table: GSEA Results Summary

  

Fig 1: Enrichment plot: HALLMARK\_MTORC1\_SIGNALING      
 Profile of the Running ES Score & Positions of GeneSet Members on the Rank Ordered List

  

| PROBE | GENE SYMBOL | GENE\_TITLE | RANK IN GENE LIST | RANK METRIC SCORE | RUNNING ES | CORE ENRICHMENT || 1 | MCM4 |  |  | 17 | 1.815 | 0.0226 | No |
| 2 | DHFR |  |  | 18 | 1.795 | 0.0483 | No |
| 3 | RRM2 |  |  | 34 | 1.660 | 0.0691 | No |
| 4 | CCNF |  |  | 158 | 1.250 | 0.0632 | No |
| 5 | MCM2 |  |  | 238 | 1.130 | 0.0642 | No |
| 6 | BUB1 |  |  | 277 | 1.084 | 0.0723 | No |
| 7 | CDKN1A |  |  | 363 | 0.993 | 0.0701 | No |
| 8 | STC1 |  |  | 433 | 0.920 | 0.0700 | No |
| 9 | GMPS |  |  | 500 | 0.865 | 0.0696 | No |
| 10 | PLK1 |  |  | 526 | 0.850 | 0.0770 | No |
| 11 | CXCR4 |  |  | 550 | 0.830 | 0.0844 | No |
| 12 | CDC25A |  |  | 631 | 0.782 | 0.0801 | No |
| 13 | UNG |  |  | 703 | 0.745 | 0.0771 | No |
| 14 | CD9 |  |  | 738 | 0.728 | 0.0810 | No |
| 15 | AURKA |  |  | 761 | 0.718 | 0.0870 | No |
| 16 | TUBG1 |  |  | 776 | 0.712 | 0.0944 | No |
| 17 | ELOVL6 |  |  | 925 | 0.649 | 0.0752 | No |
| 18 | TMEM97 |  |  | 963 | 0.636 | 0.0772 | No |
| 19 | SLC37A4 |  |  | 1195 | 0.564 | 0.0408 | No |
| 20 | TM7SF2 |  |  | 1273 | 0.539 | 0.0336 | No |
| 21 | RPA1 |  |  | 1307 | 0.530 | 0.0348 | No |
| 22 | PIK3R3 |  |  | 1327 | 0.526 | 0.0387 | No |
| 23 | RPN1 |  |  | 1418 | 0.499 | 0.0285 | No |
| 24 | FDXR |  |  | 1688 | 0.438 | -0.0170 | No |
| 25 | GGA2 |  |  | 1730 | 0.431 | -0.0188 | No |
| 26 | FADS2 |  |  | 2155 | 0.367 | -0.0951 | No |
| 27 | NUP205 |  |  | 2169 | 0.366 | -0.0924 | No |
| 28 | VLDLR |  |  | 2236 | 0.358 | -0.1000 | No |
| 29 | PFKL |  |  | 2241 | 0.358 | -0.0957 | No |
| 30 | ADIPOR2 |  |  | 2360 | 0.341 | -0.1135 | No |
| 31 | HPRT1 |  |  | 2404 | 0.334 | -0.1170 | No |
| 32 | GSK3B |  |  | 2854 | 0.284 | -0.1994 | No |
| 33 | MTHFD2L |  |  | 3113 | 0.259 | -0.2454 | No |
| 34 | ATP6V1D |  |  | 3222 | -0.254 | -0.2625 | No |
| 35 | PGK1 |  |  | 3286 | -0.263 | -0.2709 | No |
| 36 | PSMC6 |  |  | 3338 | -0.274 | -0.2768 | No |
| 37 | GOT1 |  |  | 3406 | -0.283 | -0.2857 | No |
| 38 | POLR3G |  |  | 3504 | -0.300 | -0.3001 | No |
| 39 | ERO1A |  |  | 3506 | -0.300 | -0.2960 | No |
| 40 | LTA4H |  |  | 3584 | -0.312 | -0.3063 | No |
| 41 | PSMC4 |  |  | 3608 | -0.317 | -0.3062 | No |
| 42 | BHLHE40 |  |  | 3620 | -0.318 | -0.3038 | No |
| 43 | ACTR2 |  |  | 3661 | -0.325 | -0.3069 | No |
| 44 | IDI1 |  |  | 3680 | -0.328 | -0.3057 | No |
| 45 | PSMD13 |  |  | 3687 | -0.329 | -0.3021 | No |
| 46 | NFIL3 |  |  | 3700 | -0.331 | -0.2997 | No |
| 47 | PSAT1 |  |  | 3732 | -0.336 | -0.3009 | No |
| 48 | RAB1A |  |  | 3776 | -0.345 | -0.3042 | No |
| 49 | ATP5MC1 |  |  | 3899 | -0.367 | -0.3225 | No |
| 50 | HSPA9 |  |  | 3909 | -0.369 | -0.3190 | No |
| 51 | ITGB2 |  |  | 3948 | -0.377 | -0.3209 | No |
| 52 | ETF1 |  |  | 4072 | -0.403 | -0.3388 | Yes |
| 53 | TXNRD1 |  |  | 4077 | -0.404 | -0.3338 | Yes |
| 54 | SQLE |  |  | 4082 | -0.405 | -0.3288 | Yes |
| 55 | SHMT2 |  |  | 4131 | -0.417 | -0.3321 | Yes |
| 56 | LDHA |  |  | 4145 | -0.421 | -0.3286 | Yes |
| 57 | DDIT4 |  |  | 4182 | -0.432 | -0.3294 | Yes |
| 58 | YKT6 |  |  | 4183 | -0.432 | -0.3232 | Yes |
| 59 | HSPA4 |  |  | 4206 | -0.442 | -0.3211 | Yes |
| 60 | MTHFD2 |  |  | 4212 | -0.444 | -0.3158 | Yes |
| 61 | MAP2K3 |  |  | 4216 | -0.445 | -0.3100 | Yes |
| 62 | PPA1 |  |  | 4219 | -0.445 | -0.3040 | Yes |
| 63 | HMGCR |  |  | 4221 | -0.446 | -0.2978 | Yes |
| 64 | BCAT1 |  |  | 4314 | -0.476 | -0.3087 | Yes |
| 65 | SKAP2 |  |  | 4381 | -0.495 | -0.3144 | Yes |
| 66 | STARD4 |  |  | 4393 | -0.499 | -0.3094 | Yes |
| 67 | SEC11A |  |  | 4406 | -0.503 | -0.3045 | Yes |
| 68 | EBP |  |  | 4441 | -0.517 | -0.3037 | Yes |
| 69 | SLC7A5 |  |  | 4513 | -0.547 | -0.3095 | Yes |
| 70 | PSMG1 |  |  | 4573 | -0.574 | -0.3127 | Yes |
| 71 | UFM1 |  |  | 4598 | -0.588 | -0.3089 | Yes |
| 72 | PNO1 |  |  | 4617 | -0.602 | -0.3038 | Yes |
| 73 | HMGCS1 |  |  | 4646 | -0.616 | -0.3004 | Yes |
| 74 | PSMA3 |  |  | 4650 | -0.618 | -0.2922 | Yes |
| 75 | EIF2S2 |  |  | 4683 | -0.635 | -0.2892 | Yes |
| 76 | P4HA1 |  |  | 4689 | -0.641 | -0.2811 | Yes |
| 77 | USO1 |  |  | 4699 | -0.644 | -0.2736 | Yes |
| 78 | HSPA5 |  |  | 4714 | -0.652 | -0.2670 | Yes |
| 79 | SLC2A1 |  |  | 4739 | -0.671 | -0.2620 | Yes |
| 80 | ACTR3 |  |  | 4759 | -0.684 | -0.2559 | Yes |
| 81 | NAMPT |  |  | 4906 | -0.820 | -0.2723 | Yes |
| 82 | SLC2A3 |  |  | 4942 | -0.859 | -0.2668 | Yes |
| 83 | G6PD |  |  | 4945 | -0.861 | -0.2549 | Yes |
| 84 | PPP1R15A |  |  | 4966 | -0.882 | -0.2461 | Yes |
| 85 | IGFBP5 |  |  | 4978 | -0.897 | -0.2355 | Yes |
| 86 | ME1 |  |  | 4982 | -0.899 | -0.2232 | Yes |
| 87 | TUBA4A |  |  | 4987 | -0.909 | -0.2110 | Yes |
| 88 | ENO1 |  |  | 4999 | -0.930 | -0.1998 | Yes |
| 89 | SYTL2 |  |  | 5001 | -0.932 | -0.1867 | Yes |
| 90 | CYP51A1 |  |  | 5037 | -1.013 | -0.1790 | Yes |
| 91 | SERPINH1 |  |  | 5059 | -1.050 | -0.1681 | Yes |
| 92 | SQSTM1 |  |  | 5099 | -1.149 | -0.1592 | Yes |
| 93 | PRDX1 |  |  | 5104 | -1.163 | -0.1433 | Yes |
| 94 | GLRX |  |  | 5121 | -1.212 | -0.1291 | Yes |
| 95 | NUPR1 |  |  | 5123 | -1.217 | -0.1119 | Yes |
| 96 | SLC1A5 |  |  | 5145 | -1.294 | -0.0975 | Yes |
| 97 | TRIB3 |  |  | 5148 | -1.313 | -0.0792 | Yes |
| 98 | XBP1 |  |  | 5165 | -1.393 | -0.0624 | Yes |
| 99 | DDIT3 |  |  | 5180 | -1.461 | -0.0442 | Yes |
| 100 | SLC7A11 |  |  | 5260 | -2.181 | -0.0283 | Yes |
| 101 | SLC9A3R1 |  |  | 5271 | -2.439 | 0.0046 | Yes |
Table: GSEA details [plain text format]

  

Fig 2: HALLMARK\_MTORC1\_SIGNALING: Random ES distribution      
 Gene set null distribution of ES for **HALLMARK\_MTORC1\_SIGNALING**
